# Antioxidant Protective Effect of Honey in Cigarette Smoke-Induced Testicular Damage in Rats

**DOI:** 10.3390/ijms12095508

**Published:** 2011-08-29

**Authors:** Mahaneem Mohamed, Siti Amrah Sulaiman, Hasnan Jaafar, Kuttulebbai Nainamohamed Salam Sirajudeen

**Affiliations:** 1 Department of Physiology, School of Medical Sciences, Universiti Sains Malaysia, 16150 Kubang Kerian, Kelantan, Malaysia; 2 Department of Pharmacology, School of Medical Sciences, Universiti Sains Malaysia, 16150 Kubang Kerian, Kelantan, Malaysia; E-Mail: sbsamrah@kb.usm.my; 3 Department of Pathology, School of Medical Sciences, Universiti Sains Malaysia, 16150 Kubang Kerian, Kelantan, Malaysia; E-Mail: hasnan@kb.usm.my; 4 Department of Chemical Pathology, School of Medical Sciences, Universiti Sains Malaysia, 16150 Kubang Kerian, Kelantan, Malaysia; E-Mail: sirajuden@kb.usm.my

**Keywords:** cigarette smoke, honey, testis, histology, oxidative stress

## Abstract

Cigarette smoke (CS) can cause testicular damage and we investigated the possible protective effect of honey against CS-induced testicular damage and oxidative stress in rats. CS exposure (8 min, 3 times daily) and honey supplementation (1.2 g/kg daily) were given for 13 weeks. Rats exposed to CS significantly had smaller seminiferous tubules diameter and epithelial height, lower Leydig cell count and increased percentage of tubules with germ cell loss. CS also produced increased lipid peroxidation (TBARS) and glutathione peroxidase (GPx) activity, as well as reduced total antioxidant status (TAS) and activities of superoxide dismutase (SOD) and catalase (CAT). However, supplementation of honey significantly reduced histological changes and TBARS level, increased TAS level, as well as significantly restored activities of GPx, SOD and CAT in rat testis. These findings may suggest that honey has a protective effect against damage and oxidative stress induced by CS in rat testis.

## 1. Introduction

Cigarette smoking has been shown to be associated with abnormalities in male reproductive function such as decreased sperm count [1] and motility [2] as well as increased percentage of morphologically abnormal sperm [3] and sperm chromatin damage [4,5]. In experimental studies, rodents that are exposed to cigarette smoke (CS) have been reported to have degenerated and lower number of Leydig cells [6] as well as significant decrease in germ cell count and seminiferous tubule diameter in testis [7,8]. The exact mechanisms leading to the detrimental effects on sperm parameters and testis are not clearly understood. However, it has been postulated that CS produces oxidative stress as there is a significantly higher level of malonaldehyde, a marker of lipid peroxidation, in rat testis after 45 days of exposure to CS as compared to controls. This is also associated with a significantly lower glutathione level and activity of glutathione peroxidase in rat testis [9].

Honey is a natural product of honey bees formed from nectar collected from blossoms. It has been reported that honey contains moisture and carbohydrates including sugars such as fructose and glucose [10]. It also contains enzymes such as catalase and glutathione reductase, minerals such as iron and zinc, vitamins such as vitamins A and E as well as phenolic compounds and organic acids [11–15]. Scientific studies have shown that honey possesses some biological properties such as antimicrobial [16,17], anti-inflammatory [18,19] and antioxidant [20–22] properties. Recently, honey at the dose of 1.2 g/kg/day has been shown to improve sperm parameters and serum testosterone level in rats exposed to CS for 13 weeks [23]. It is postulated that honey could improve the impaired testicular function by possibly reducing the testicular damage and oxidative stress. Therefore, the aim of this study was to determine the possible protective effect of honey against the testicular damage and oxidative stress in rats exposed to CS.

## 2. Results and Discussion

### 2.1. Histopathology of Testis

The histopathological findings of rat testis, such as seminiferous tubule diameter and epithelial height, percentage of tubule with germ cell loss and Leydig cell count from all experimental groups are shown in Table 1. Testis in rats from control (given distilled water) and H groups (given honey) that were exposed to room air, had normal histological findings. CS significantly reduced seminiferous tubule diameter and epithelial height, and Leydig cell count, as well as significantly increased the percentage of tubule with germ cell loss in rats from CS group, as compared to control and H groups. In contrast, these parameters were found to be significantly improved in rats supplemented with honey and exposed to CS (H + CS group).

**Figure 1:**
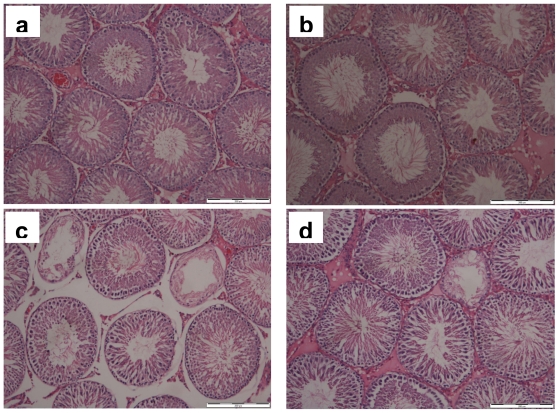
Representative photomicrographs of hematoxylin and eosin staining of the testis (scale bar: 200 μm) showing the seminiferous tubules from control group that received distilled water and exposed to room air (**a**); H group that received honey and exposed to room air (**b**); CS group that received distilled water and exposed to cigarette smoke (**c**); and H + CS group that received honey and exposed to cigarette smoke (**d**).

Figure 1 shows representative photomicrographs of testicular sections showing the seminiferous tubules from all the experimental groups. Normal morphological structures of seminiferous tubules and germ cells were observed in control and H groups. The section from CS group showed a presence of smaller tubules with germ cell loss. However, the section from H+CS group had less damage to the tubules and germ cells as compared to CS group.

**Figure 2:**
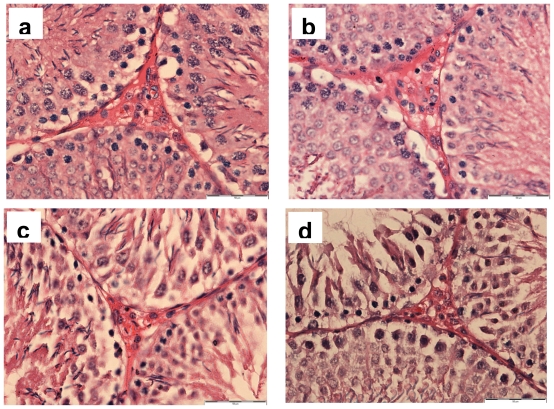
Representative photomicrographs of hematoxylin and eosin staining of the testis (scale bar: 50 μm) showing Leydig cells in intertubular space from control group that received distilled water and exposed to room air (**a**); H group that received honey and exposed to room air (**b**); CS group that received distilled water and exposed to cigarette smoke (**c**); and H + CS group that received honey and exposed to cigarette smoke (**d**).

Figure 2 shows representative photomicrographs of testicular sections showing Leydig cells in intertubular space from all the experimental groups. Normal feature and number of Leydig cells were observed in control and H groups. The section from CS group showed the presence of degenerated and reduced number of Leydig cells. However, the section from H + CS showed less damage to the Leydig cells as compared to CS group.

### 2.2. Biochemical Analyses

The findings on oxidative stress markers in testis from all experimental groups are shown in Table 2. There were significantly increased lipid peroxidation (thiobarbituric acid reactive substance [TBARS]) with reduced total antioxidant status (TAS) and superoxide dismutase (SOD) and catalase (CAT) activities in rats from CS group as compared with control and H group. The activity of glutathione peroxidase (GPx) in CS group was significantly increased than control group. However, with the supplementation of honey in rats from H + CS, these parameters were significantly improved while total glutathione level was significantly increased than control and CS groups. Moreover, the level of TAS in H group was significantly increased than other experimental groups.

The findings on *in vitro* antioxidant capacities of honey used in this study are shown in Table 3. Based on the total phenolic content, Ferric Reducing Antioxidant Power (FRAP) and 1,1-diphenyl-2-picrylhydrazil (DPPH) assays, it was found that this honey had antioxidant capacities.

In this study, CS group had decreased seminiferous tubule diameter and epithelial height, decreased number of Leydig cell and increased percentage of seminiferous tubule with germ cell loss as compared to control group which are in agreement with other studies [6–8] suggesting the presence of testicular degeneration due to the toxic effect of CS on the structures of testis. However, with the supplementation of honey, these changes were significantly reduced suggesting that honey has a protective effect against the toxic effect of CS on the structures of testis in rats. Moreover, this finding on a decrease in CS-induced toxicity on the testicular structures might explain our previous findings on the improved sperm production and testosterone level in rats with honey supplementation [23].

The level of TBARS, the lipid peroxidation end product that indicates oxidant activity, in rats from CS group was significantly increased compared with control group. This might indicate that CS exposure for 13 weeks used in this study produced higher oxidant activity or oxidative stress causing lipid peroxidation in rat testicular tissue. This finding corresponds to the earlier studies when the rats were exposed to CS for 45 [9] and 60 days [24]. The higher oxidant activity could be produced by CS as CS has been reported to have free radicals such as nitric oxide (NO), quinone, semiquinone, hydroquinone and carbon-centred radicals such as acyl- and alkylaminocarbonyl radicals [25,26]. NO can then be oxidized to form nitrogen dioxide (NO_2_) while quinone, semiquinone and hydroquinone can be oxidized to form superoxide anion which eventually forms other reactive oxygen species (ROS) including hydrogen peroxide and hydroxyl radical [25]. Testicular cells and sperm are susceptible to oxidative damage by free radicals as their plasma membrane contains abundance of polyunsaturated fatty acids [27]. Lipid peroxidation of the cellular membrane may eventually result in dysfunction and structural damage of the cell [28]. Therefore, the abnormalities observed in the testicular structures including germ and Leydig cells found in this study might be attributed to the peroxidation of polyunsaturated fatty acids in their plasma membranes by CS.

There was also a concomitant statistically significant reduced level of TAS, which measures the synergistic interaction of all the antioxidants including both enzymatic and non-enzymatic antioxidants [29], in rat testis from CS group. This reduced TAS might indicate the presence of low antioxidant capacity in scavenging ROS, preventing the formation of free radicals and/or terminating free radical chain reactions [28] resulting in testicular cells damage as observed in rat testis exposed to CS. However, with the supplementation of honey, TBARS level was significantly reduced and TAS level was significantly increased than CS group but similar with control group. These findings might suggest that honey might have some protective effect on the oxidative stress in rat testis exposed to CS. This protective effect, therefore, might provide the possible explanation for the improved findings on histological features in rats from H+CS group.

Antioxidant enzymes are essential part of the cellular defense against ROS. The activities of antioxidant enzymes such as SOD (converts superoxide anion to hydrogen peroxide and oxygen) and CAT (reduces or scavenges hydrogen peroxide to form water and oxygen) [28], were significantly reduced in rats from CS group. The reduced activities of these two main antioxidant enzymes might partly contribute to the reduced TAS level found in CS group. The imbalance between ROS generation and scavenging activities may induce oxidative stress which in turn may induce oxidative damage to the cellular component and altered many cellular functions including loss of enzymatic activity [30]. Therefore, it is suggested that the reduced SOD and CAT activities might be due to reduced enzyme synthesis by the damage testicular cells or inactivation of the enzymes by the ROS. However, with the supplementation of honey, the activities of SOD and CAT in rats from H + CS group were significantly restored. Thus, it is suggested that honey might increase the bioavailability of SOD and CAT possibly by reducing the damage to the testicular cells as found in this study. The increased SOD activity subsequently might scavenge the excess amount of superoxide anion in the rat testis when exposed to CS. Furthermore, the increased CAT activity could also be as a compensatory mechanism to scavenge high hydrogen peroxide formed by the concomitant increased SOD activity in rats from H + CS group.

On the other hand, the activity of GPx, which involves in conversion of hydrogen peroxide to form water and oxidized glutathione [28], was significantly increased in rats from CS group. It is suggested that CS up-regulates or increases the synthesis of GPx to reduce hydrogen peroxide which is produced by CS [25] and more glutathione would be oxidized in this reaction [28]. This might result in a low level of glutathione (reduced form) which in turn might stimulate the synthesis of glutathione [31] to replenish the glutathione level. Therefore, this normalization of glutathione level might explain the normal level of total glutathione found in rats from CS group. Oxidative stress induced by CS may also stimulate the synthesis of glutathione by up-regulating the expression of glutathione synthetic enzymes [32]. The newly synthesized glutathione, however, might be oxidized due to increased GPx activity which might alternatively explain the normal level of total glutathione in rats from CS group. On the other hand, in rats from H+CS group, the activity of GPx was restored. The normal level of GPx could be due to the concomitant increased CAT activity which reduces hydrogen peroxide produced by CS. However, the level of total glutathione in rats from H + CS group was significantly increased compared to the other experimental groups. The increased total glutathione level could be due to increased glutathione synthesis stimulated by oxidative stress [32] in the testis induced by CS. Furthermore, the normal activity of GPx might further contribute to the increased total glutathione in rats from H + CS group.

Biochemical *in vitro* studies on this honey had shown that it had *in vitro* antioxidant capacities. Phenolic compounds have been suggested to be the main antioxidants in honey as highly significant correlations between total phenolic content and antioxidant and antiradical activities of honey have been reported [20]. Phenolic compounds are phytochemicals present in plants including fruits and vegetables that have antioxidant properties [33]. Moreover, the antioxidant and radical scavenging activity of honey in a cultured endothelial cell line subjected to oxidative stress are mainly contributed by the phenolic acids and flavonoids in honey [34]. Phenolic compounds may protect oxidative stress by directly neutralizing reactive oxidants via its electron- or hydrogen-donating capacity, which would otherwise oxidize cellular components including lipid, protein and nucleic acid [33]. Therefore, it is plausible to suggest that phenolic compounds present in honey might directly neutralize ROS and subsequently partly attributed to the reduced lipid peroxidation and increased activities of antioxidant enzymes such as SOD, CAT and GPx in rats from H + CS group. Phenolic compounds, namely, flavonoids have also been found to increase the expression of γ-glutamylcysteine synthetase, a rate limiting enzyme for the synthesis of glutathione in both *in vitro* and *in vivo* studies [33]. Consequently, it is also possible that phenolic compounds present in honey might act indirectly by increasing expression of γ-glutamylcysteine synthetase resulting in increased total glutathione level in rats from H + CS group. Nevertheless, other antioxidants such as vitamins A and E [13] and catalase [11] together with phenolic compounds in honey might possibly interact synergistically to produce protection in the testis against oxidative stress in rats exposed to CS. Thus, the honey with its antioxidant capacities might counteract the changes observed in rat testicular oxidative stress markers and this partly attribute to the reduced detrimental effects of CS on the testicular structures observed in this study. However, there is a need to further determine the constituent of this honey that is responsible to its antioxidant effect.

## 3. Experimental Section

### 3.1. Chemicals

Honey used in this study is a wild multifloral honey supplied by Federal Agricultural Marketing Authority (FAMA), Malaysia. It was collected from beehives built on a tall tree, *Koompassia excelsa* (locally named as ‘Tualang’ tree) that grows in the Rain Forest of Kedah, Malaysia and this honey is also locally known as Tualang honey. The composition of this honey is presented as follows: total reducing sugar (68.30%) [fructose (39.84%), glucose (25.29%), maltose (3.17%), fructose/glucose ratio (1.58)], sucrose (0.87%) and 5-hydroxymethyl-2-furfural (4.19 mg per kg). Commercially available cigarettes (Benson & Hedges, British American Tobacco Bhd., Malaysia) containing about 1.4 mg nicotine and 15 mg tar per cigarette were used for all CS exposures. All chemicals and reagents used in this study were of analytical grade.

### 3.2. Animals

Thirty-two adult male Sprague-Dawley rats, aged 10 weeks (270–320 g) were obtained from Laboratory Animal Research Unit, Health Campus, Universiti Sains Malaysia. Rats were individually housed in a polycarbonate cage and maintained on a 12-h light/dark cycle at 20–24 °C and provided a standard pellet diet and water *ad libitum*. This study protocol was approved by the Animal Ethics Committee, Universiti Sains Malaysia (PPSG/07(A)/044/2007[32]) and the animals were handled in accordance with the Guide for the Care and Use of Laboratory Animals by National Institute of Health.

### 3.3. Experimental Design

Animals were randomly divided into 4 groups of 8 each as follows: control, honey (H), CS and H plus CS (H + CS) groups. Rats in control and CS groups received oral administration of distilled water (0.5 mL) while rats in H and H + CS groups received freshly prepared honey orally by gavage daily at a dose of 1.2 g/kg body weight (using distilled water as a vehicle). This dose was worked out relative to the local human consumption of honey which is 0.2 g/kg body weight daily. Animals in CS and H+CS groups were exposed to CS for 3 times daily using a whole body smoke exposure chamber (45 × 25 × 20 cm with 2 compartments). For each CS exposure, the smoke produced from 10 burning cigarettes in one compartment was continuously ventilated by 2 air pumps to another compartment where the rats were placed and exposed to the smoke for 8 min [35]. The rats in control and H groups were subjected to the similar condition but exposed to room air. After 13 weeks of treatment, rats were sacrificed under over exposure to diethyl ether and testes were carefully dissected for histopathological and oxidative stress markers analyses. Antioxidant capacities of the honey were also determined *in vitro* in this study.

### 3.4. Histopathological Examination

The testis was fixed in Bouin’s fixative, dehydrated and embedded in paraffin blocks. Each tissue block was sectioned into 5 μm thickness, stained with hematoxylin and eosin and evaluated histologically [36] using a light microscope and an image analyzer (Soft Imaging System, VGA Utilities version 3.67e). For each rat, testicular tissue section was assessed for any possible histological changes such as desloughing of germ cells, disorganization of the tubular elements, presence of multinucleated giant cell or germ cell loss (either focal, generalized or both) in 200 seminiferous tubules at 100× magnification. Seminiferous tubular diameter and seminiferous epithelial height of 20 random round seminiferous tubules were measured [37] and number of Leydig cell of 20 random intertubular areas (each intertubular area was surrounded by three seminiferous tubules) was counted under light microscope at 40× and 400× magnifications, respectively.

### 3.5. Biochemical Analysis

#### 3.5.1. Oxidative Stress Markers Assays in Rat Testis

Testicular tissue was weighed and homogenized to make 10% homogenate (w/v) in ice-cold 0.1 M Tris (hydroxymethy) aminomethane-HCl (Tris-HCl), pH 7.4, using an ice-chilled glass-homogenizing vessel in a homogenizer fitted with Teflon pestle (Glas-Col, USA). The homogenate was then centrifuged in a refrigerated centrifuge (Eppendorf 5810R, Germany) at 1000 × g at 4 °C for 15 min to remove nuclei and debris. The supernatant obtained was used as a sample for the following assays: total protein, lipid peroxidation, TAS, total glutathione and activities of enzymatic antioxidants.

Protein concentration was estimated by Micro TP kit (Wako Pure Chemicals, Osaka, Japan) with bovine serum albumin as a standard according to the method of Watanabe *et al.* [38]. Briefly, 0.01 mL of sample or standard was added to 1 mL of Micro TP reagent in duplicate. The absorbance for sample and standard were measured spectrophotometrically at 600 nm. Lipid peroxidation measurement was determined as measurement of TBARS according to the method of Ohkawa *et al.* [39]. 1,1,3,3-tetraethoxypropane, a precursor of malonaldehyde (MDA) was used as a standard for this assay. The amount of TBARS formed was measured spectrophotometrically at 532 nm and expressed as nmol of MDA equivalent per mg protein. TAS was measured by the reaction of antioxidants in the sample with a definite amount of exogenously provided hydrogen peroxide according to the method of Koracevic *et al.* [40] using uric acid as a standard. In this assay, the reaction of Fe-EDTA complex with hydrogen peroxide formed hydroxyl radicals. These hydroxyl radicals degraded benzoate which in turn leading to the release of TBARS. Antioxidants from the sample caused suppression of the TBARS production, which was proportional to its antioxidants concentration. The reaction was measured spectrophotometrically at 532 nm and TAS was expressed as μmol of uric acid equivalent per mg protein.

SOD activity was assayed using a commercially available kit, Superoxide Dismutase Assay Kit II (EMD Chemicals, Gibbstown, NJ) which utilizes a tetrazolium salt for detection of superoxide radicals generated by xanthine oxidase and hypoxanthine. The SOD activity in each sample was expressed as unit per mg protein. One unit of SOD activity was defined as the amount of enzyme needed to exhibit 50% dismutation of the superoxide radical. CAT activity was determined according to the method of Goth [41] using hydrogen peroxide as a substrate. The enzyme decomposed the hydrogen peroxide and the remaining hydrogen peroxide was then reacted with molybdate ions to form a yellowish complex, which had a maximum absorbance at 405 nm. CAT activity in each sample was expressed as unit per mg protein. One unit of CAT was defined as the amount of enzyme that catalyzes the decomposition of 1 μmol of hydrogen peroxide per min.

Total glutathione was measured using Glutathione Assay kit (Sigma-Aldrich, St. Louis, MO, USA). In this assay, reduced glutathione (GSH) caused reduction of 5,5′-dithiobis-2-nitrobenzoic acid (DTNB) to produce 5-thio-2-nitrobenzoic acid (TNB) and oxidized glutathione (GSSG). Then GR catalyzed the reduction of GSSG in the presence of nicotinamide adenine dinucleotide (NADPH), which was oxidized to NADP^+^. The absorbance due to the formation of TNB was measured spectrophotometrically at 412 nm and GSH was used as a standard. Total glutathione in each sample was expressed as nmol of GSH equivalent per mg protein. GPx activity was estimated using hydrogen peroxide as a substrate according to the method of Luchese *et al.* [42]. GPx catalyzed the oxidation of GSH by hydrogen peroxide. In the presence of glutathione reductase and NADPH, GSSG was immediately converted to the reduced form with a concomitant oxidation of NADPH to NADP^+^. The decrease in the concentration of NADPH was measured at 340 nm. GPx activity was expressed as unit per mg protein and one unit of GPx was defined as the amount of enzyme that catalyzed the oxidation of one nmol of NADPH per min.

GR activity was assayed using GSSG as a substrate. GR catalyzed the reduction of GSSG in the presence of NADPH, which was concomitantly oxidized to NADP^+^. The decrease in absorbance due to decreased concentration of NADPH was measured at 340 nm. GR activity was expressed as unit per mg protein and one unit of GR was defined as the amount of enzyme that catalyzed the oxidation of 1 nmol of NADPH per min [42]. GST activity was assayed using 1-chloro-2,4-dinitrobenzene (CDNB) as a substrate. Conjugation of GSH with CDNB was measured spectrophotometrically at 340 nm. GST activity was expressed as unit per mg protein and one unit of GST was defined as the amount of enzyme that catalyzed the conjugation of 1 nmol of GSH-CDNB per min [43].

#### 3.5.2. Determination of the Antioxidant Capacities of Honey

Total phenolic content in honey was determined using Folin-Ciocalteu’s phenol reagent according to the method of Beretta *et al*. [44]. The phenolic compound present in honey reduced the Folin-Ciocalteu reagent to form blue colour chromogens which measured spectrophotometrically at 750 nm. Gallic acid was used as a standard and total phenolic content was expressed as μmol of gallic acid equivalent per kg of honey.

FRAP assay to determine the antioxidant activity of honey was done according to the method of Benzie *et al.* [45]. The antioxidant present in honey reduced ferric tripyridyltriazine (Fe^3+^-TPTZ) complex to ferrous tripyridyltriazine (Fe^2+^-TPTZ) complex which was measured spectrophotometrically at 593 nm. Ferrous sulfate was used as a standard and the reducing ability of honey was expressed as μmol of Fe^2+^ equivalent per liter.

Free radical scavenging activity was measured using 1,1-dphenyl-2-picrylhydrazil (DPPH) assay as reported earlier [46,47] with minor modifications. Briefly, 0.75 mL of the honey solution (10%, w/v) was mixed with 1.5 mL of 0.09 mg/mL DPPH in methanol in a test tube. The mixture was then incubated at 25 °C for 5 min after which the absorbance was measured spectrophotometrically at 517 nm against a test blank (honey solution with 1.5 mL of distilled water:methanol). The absorbance of 1.5 mL of DPPH radical control was measured with 0.75 mL of distilled water:methanol against DPPH radical control blank (2.25 mL of distilled water:methanol). The antiradical activity was expressed as percentage inhibition of DPPH radical by honey.

### 3.6. Statistical Analyses

Statistical analyses were carried out using the Statistical Package for Social Science (SPSS) version 12.0.1. Data with normal distribution and homogenous variance were analyzed using One-way analysis of variance (ANOVA) followed by Tukey *post-hoc* test and presented as mean ± standard deviation. Meanwhile, data with non-normal distribution and non-homogenous variance were analyzed by Kruskal-Wallis test followed by Mann-Whitney *U* test and presented as median (interquartile range). A value of *p* < 0.05 was considered to be statistically significant.

## 4. Conclusions

These findings demonstrated that honey significantly reduced the toxic effects of CS on the testicular structures. It also reduced oxidative stress by reducing lipid peroxidation and restoring antioxidant system in rat testis. Hence, this study might suggest that honey has a protective effect against the histological changes and oxidative stress induced by CS in rat testis. This effect is probably through its various nutritional constituents due, at least in part, to their synergistic antioxidant property. Further studies are suggested to evaluate vacuolation and necrosis score as well as apoptotic index by TUNEL staining in order to have a clearer and more complete picture on the effects of honey supplementation on CS-induced histological changes in the rat testis. Further studies are also needed to elucidate the exact molecular mechanism of action of this honey and its constituent as a natural antioxidant in counteracting oxidative stress-induced testicular toxicity. The possible beneficial role of honey supplementation, either alone or in combination with other drugs, in protecting or treating male reproductive health problems particularly related to oxidative stress is suggested to be further studied.

## Figures and Tables

**Table 1:** Testicular morphometry, percentage of seminiferous tubule with germ cell loss and Leydig cell count in rat testis from all experimental groups.

Parameters	Control Group	H Group	CS Group	H + CS Group	*p* (KW test)
Seminiferous tubule diameter (μm)	276.32 (255.22–286.97)	260.74 (256.94–275.27)	246.24 (237.19–256.50)^a^,^b^	267.46 (258.72–283.21)^c^	<0.05
Seminiferous epithelial height (μm)	81.80 (76.05–90.26)	78.60 (76.39–83.17)	71.01 (66.95–74.82)^a^,^b^	77.47 (73.19–80.79)^c^	<0.05
Seminiferous tubule with germ cell loss (%)	0.00 (0.00–1.00)	0.00 (0.00–0.63)	7.50 (4.88–15.50)^a^,^b^	2.25 (2.25–5.13)^a^,^b^,^c^	<0.001
Leydig cell count	206.5 (197.8–219.0)	195.5 (178.8–219.0)	154.5 (138.5–162.5)^a^,^b^	175.0 (165.3–190.3)^a^,^c^	<0.01

Data are presented as median (interquartile range) (*n* = 6 per group). H, honey; CS, cigarette smoke; H + CS, honey plus CS; KW, Kruskal-Wallis.

a *p* < 0.05 compared with control group;

b *p* < 0.05 compared with H group and

c *p* < 0.05 compared with CS group by Mann-Whitney *U* test.

**Table 2:** Oxidative stress markers in rat testis from all experimental groups.

Parameters	Control group	H group	CS group	H + CS group	*p*
TBARS (nmol MDA Eq/mg protein)#	0.29 (0.26–0.41)	0.34 (0.19–0.49)	0.68 (0.54–0.87)^a^,^b^	0.32 (0.27–0.41)^c^	<0.01£
TAS (μmol uric acid Eq/mg protein) @	0.23 ± 0.02	0.26 ± 0.02 a	0.14 ± 0.03 a,b	0.19 ± 0.03 b,c	<0.001 §
SOD activity (unit/mg protein) #	1.30 (1.21–1.43)	1.23 (0.89–1.31)	0.82 (0.68–0.94) a,b	1.09 (0.95–1.51) c	<0.01 £
CAT activity (unit/mg protein) #	20.54 (17.58–26.52)	25.25 (22.51–28.44)	16.30 (14.38–19.21) a,b	20.21 (19.06–22.64)^b^,^c^	<0.01 £
Total glutathione (nmol GSH Eq/mg protein) @	1.45 ± 0.14	1.46 ± 0.34	1.40 ± 0.33	1.93 ± 0.50 a,c	<0.05 ^$^
GPx activity (unit × 10^3^/mg protein) #	166.56 (161.45–174.22)	168.41 (153.41–195.44)	183.69 (179.83–195.67) a	163.26 (156.38–170.99) c	<0.05 £
GR activity (unit/mg protein) @	20.64 ± 1.39	20.50 ± 2.63	20.29 ± 1.53	21.31 ± 1.17	NS §
GST activity (unit × 10^3^/mg protein) #	0.75 (0.67–0.80)	0.73 (0.68–0.89)	0.66 (0.63–0.71)	0.70 (0.68–0.74)	NS £

Data are presented as

# median (interquartile range) and

@ mean ±standard deviation (*n* = 8 per group). H, honey; CS, cigarette smoke; H + CS, honey plus CS; Eq, equivalent; TBARS, thiobarbituric acid reactive substance; MDA, malonaldehyde; TAS, total antioxidant status; SOD, superoxide dismutase; CAT, catalase; GSH, glutathione; GPx, glutathione peroxidase; GR, glutathione reductase; GST, glutathione-S-transferase; NS, not significant;

£ Kruskal-Wallis test followed by Mann-Whitney *U* test;

§ One-way analysis of variance followed by Tukey multiple comparison *post-hoc* test;

a *p* < 0.05 compared with control group,

b *p* < 0.05 compared with H group and

c *p* < 0.05 compared with CS group.

**Table 3:** Antioxidant capacities of honey.

**Parameters**	
Total phenolic content	219.53 ± 8.98 mg of gallic acid Eq/kg of honey
Antioxidant activity (FRAP assay)	369.79 ± 21.46 μmol of Fe^2+^ Eq/L
Free radical scavenging activity (DPPH assay)	47.25 ± 0.62% inhibition of DPPH radical

Data are mean ± standard deviation of quadruplicate determinations. FRAP, ferric reducing antioxidant power; DPPH, 1,1-diphenyl-2-picrylhydrazil; Eq, equivalent.
